# Histological Features of Celiac-Disease-like Conditions Related to Immune Checkpoint Inhibitors Therapy: A Signal to Keep in Mind for Pathologists

**DOI:** 10.3390/diagnostics12020395

**Published:** 2022-02-03

**Authors:** Rachele Del Sordo, Umberto Volta, Vassilios Lougaris, Paola Parente, Angelo Sidoni, Mattia Facchetti, Gabrio Bassotti, Illuminato Carosi, Celeste Clemente, Vincenzo Villanacci

**Affiliations:** 1 Department of Medicine and Surgery, Section of Anatomic Pathology and Histology, Medical School, University of Perugia, 06132 Perugia, Italy; rachele.delsordo@unipg.it (R.D.S.); angelo.sidoni@unipg.it (A.S.); 2 Department of Medical and Surgical Sciences, University of Bologna, 40126 Bologna, Italy; umberto.volta@unibo.it; 3 Pediatrics Clinic, Department of Clinical and Experimental Sciences, University of Brescia and Children’s Hospital, ASST-Spedali Civili, 25123 Brescia, Italy; vlougarisbs@yahoo.com; 4 Pathology Unit, Fondazione IRCCS Ospedale Casa Sollievo della Sofferenza, 71013 San Giovanni Rotondo, FG, Italy; i.carosi@operapadrepio.it (I.C.); celesteclemente@alice.it (C.C.); 5 Institute of Pathology, ASST-Spedali Civili Brescia, 25123 Brescia, Italy; facchetti.m@gmail.com; 6 Gastroenterology & Hepatology Section, Department of Medicine and Surgery, University of Perugia, 06132 Perugia, Italy; gabassot@tin.it

**Keywords:** immune checkpoint inhibitors, celiac disease, CD-like conditions, enteropathy, duodenitis, PD-1, PDL-1, CTLA-4

## Abstract

Immune checkpoint inhibitors (ICIs) targeting cytotoxic T-lymphocyte-associated antigen 4 (CTLA-4), programmed cell death protein (PD-1), and its ligand PDL-1, are finding increasing application in the treatment of malignant neoplasms. The widespread clinical use of these drugs, however, resulted in the discovery of side effects. The occurrence of celiac disease (CD) after ICIs therapy has been reported in the literature, but its incidence remains unknown and the role of ICIs in its onset is not yet clear. In this review, we examine the published data on this topic in order to better understand and define this entity from a histological point of view. We performed an electronic literature search to identify original reports in which CD or pathological CD-like conditions were documented histologically in patients treated with ICIs. We identified ten papers. A total of twenty-five patients were included in these publications, eleven of them receiving a serologic and histological diagnosis of CD, and four a histological diagnosis of CD-like conditions, in which pathogenesis appears to be multifactorial. ICIs can cause a CD-like enteropathy and biopsies with clinical integration are crucial to diagnose this condition. CD rarely has been observed during treatment with ICIs and its morphological aspects are similar to ICIs-CD enteropathy. Moreover, the onset of ICIs-CD may have a distinct immune mechanism compared to classical CD. Thus, the pathologists must make a histological diagnosis of CD with caution and only in adequate clinical and serological context.

## 1. Introduction

Biological therapy development in inflammatory bowel disease (IBD) has allowed for the identification of several novel compounds that can interfere with distinct immunological pathways [1,2]. Among these compounds, immune checkpoint inhibitors (ICIs) targeting cytotoxic T-lymphocyte-associated antigen 4 (CTLA-4), programmed cell death protein (PD-1), and its ligand PDL-1, have been added to increasing treatment indications among patients affected by malignancies [3]. The rationale for their implementation in this setting originates from the observation that CTLA-4, PD-1, and PDL-1 are overexpressed in the tumour microenvironment, on tumour cells, and T cells intermingled with neoplastic cells [4]. It has been shown that tumour cells may escape from the immune surveillance by activating the CTLA-4B7 (B7 being the ligand of CTLA-4) and PD-1/PDL-1 checkpoint signalling pathways, which in turn suppress the CD8+ cytotoxic T-cell immune response [5]. The implementation of these specific ICIs should improve the host immune response against the tumour cells and thus achieve positive results in terms of prognosis and survival.

The Food and Drug Administration has approved several monoclonal antibodies blocking CTLA-4 (ipilimumab), PD-1 (pembrolizumab, nivolumab), and PDL-1 (atezolizumab, avelumab, durvalumab) for treatment of solid tumours, such as melanomas [6], non-small cell lung carcinoma [7], squamous cell carcinoma of the head and neck [8], urothelial carcinoma [9], gastroesophageal cancer [10], renal cell carcinoma [11], colorectal cancer with high microsatellite instability status (MSI-H) or mismatch repair deficient (MMRd) profile [12], hepatocellular carcinoma [13], classic Hodgkin’s lymphoma [14], and Merkel cell carcinoma [15].

The increasing implementation of these drugs in different settings has underlined the possibility of side effects arising in treated patients. Their interference with the immune system appears to favour the onset of autoimmune manifestations, defined as immune-related adverse events (IRAEs) [16,17]. IRAEs typically have a delayed onset and prolonged duration (months or even years after the discontinuation of treatment) and affect most commonly the gastrointestinal tract, liver, endocrine organs, lungs, and skin [18]. IRAEs affecting the gastrointestinal tract are rather frequent, mostly with anti-CTLA4 than with anti-PD-1/PDL-1 agents, and have been documented and studied both from a clinical and histological point of view [19,20,21,22]. The upper (oesophagus, stomach, and duodenum) and the lower gastrointestinal tract (small intestine and colon) may be involved in this setting, with colitis being the most frequent manifestation [23,24]. ICIs related colitis has been extensively studied and colon biopsies from affected patients show a heterogeneous spectrum of histological patterns resembling lymphocytic colitis, ulcerative colitis, or Crohn disease [22,25,26,27,28]. Moreover, a graft-versus-host-disease-like colitis (GVHD-like) with an apoptotic pattern is also well known and, recently, it has been described in a setting of Hodgkin lymphoma treated with anti-CD30 monoclonal antibodies [29]. On the other hand, the histopathological alterations in the upper gastrointestinal tract induced by ICIs have been rarely reported [23,24,30,31,32]. In particular, celiac disease (CD) or CD-like histological features in duodenal biopsies have only been observed in a very small number of patients and the role of ICIs in their onset is not clear yet. It has been hypothesized that the loss of immune tolerance induced by ICIs may unmask CD and /or CD-like conditions [33]. ICIs CD-like conditions, unlike CD arising during ICIs therapy (ICIs-CD), do not respond to gluten-free diet (GFD) but are responsive to steroids [33].

## 2. CD, CD-like Conditions, and Duodenitis

Celiac disease (CD) is an immune-mediated inflammatory disorder of the small intestine developing in genetically susceptible individuals after exposure to gluten, characterized by malabsorption resulting from mucosal injury after ingestion of wheat gluten or related rye and barley proteins [34]. CD can occur at any age, from early childhood to elderly, having a variable incidence, with a worldwide prevalence of about 1:100. A correct diagnosis of CD requires the integration of clinical, serological, genetic, and histological findings [35]. The most important serological investigations in the diagnosis of CD are IgA class antitransglutaminase antibodies (TTGA) with the highest sensitivity (98%) and specificity estimated at around 90%, and IgA class antiendomysial antibodies (EMA), this test having a lower sensitivity compared to IgA-class TTGA (90% vs. 98%) but showing an almost absolute specificity for CD. Moreover, CD is closely associated with histocompatibility antigens (HLA) DQ2 and DQ8 [36]. Endoscopic features of CD include a paucity or loss of mucosal folds, effacement of folds with oedema, presence of a mosaic pattern, scalloping, nodularity, and increased visibility of the vasculature. Finally, duodenal biopsy in CD shows characteristic histologic findings and the diagnosis and severity grading of CD include three elementary lesions: intraepithelial lymphocytosis (>25 T lymphocytes CD3 positive per 100 enterocytes), crypt hyperplasia (more than one mitotic figure per crypt, usually accompanied by a decrease in mucosecretive activity) and villous atrophy (a villus/crypt ratio lower than 3:1). The Marsh histopathological classification is universally recognized and has been extensively validated [37]. It classifies CD on the basis of presence or absence of elementary lesions, as follows: Type 1 or infiltrative lesion (only intraepithelial lymphocytosis); Type 2 or hyperplastic lesion (intraepithelial lymphocytosis and crypt hyperplasia); Type 3 or destructive lesion (intraepithelial lymphocytosis, crypt hyperplasia, and villous atrophy). Oberhuber modified the Marsh classification [38] by splitting the Type 3 lesion in three subgroups based on the extent of villous atrophy: Type 3a (mild villous atrophy), Type 3b (moderate villous atrophy); Type 3c (total villous atrophy). Further classifications have been proposed to achieve the simplification of the diagnostic criteria and reduction of the number of categories in order to increase the interobserver agreement, facilitate the clinician’s work, and improve the communication between pathologist and clinician. In this setting, the Corazza and Villanacci classification classify CD in only three categories: Type A (nonatrophic) without villous atrophy but intraepithelial lymphocytosis and with or without crypt hyperplasia; Type B (atrophic) intraepithelial lymphocytosis, crypt hyperplasia, and villous atrophy; this category was further subdivided into Type B1, moderate villous atrophy and Type B2, complete villous atrophy [39,40]. Numerous disorders can cause a damage of the intestinal mucosa with similar histological features. Some of these conditions including immunodeficiency disorders (i.e., IgA deficiency, common variable immunodeficiency, HIV enteropathy), a variety of infections, (i.e., HP, *Giardia, Cryptosporidium, Cyclospora*), and drug enteropathies (NSAIDs, recently approved antineoplastic agents, angiotensin II antagonists, immunosuppressive medications such as methotrexate, azathioprine, and mycophenolate mofetil) have rarely been associated with severe villous blunting, showing only a more or less pronounced increase of intraepithelial lymphocytes [41,42,43,44,45]. These conditions characterized by intraepithelial lymphocytosis without villous blunting fall into the category of so-called “microscopic enteritis”. Microscopic enteritis is an “umbrella term” proposed by the Bucharest Consensus [46], to indicate a group of heterogeneous conditions that represent a challenge for the gastroenterologist, and the strict collaboration between the clinician and the pathologist is mandatory to achieve a correct diagnosis [47]. In some cases, histological features such as neutrophil crypt abscess, prominent crypt apoptosis, prominent eosinophil infiltration, plasma cells absence, and crypt distortion can help in the differential diagnosis [42]. In contrast, duodenitis is a reactive condition to the injurious effect of gastric acid. Drugs such as NSAIDs, cigarettes, and alcohol can enhance the effects of gastric acid. Histologically, there are variable degrees of villous blunting, without pathological increase of T lymphocytes, gastric surface metaplasia, acute inflammation, erosions, and ulcerations. In the chronic phase, there is Brunner’s glands hyperplasia [48,49].

In this review, we summarize CD and CD-like histological features in patients with ICIs therapy reported in the literature and comment on the possible immune-related pathogenic mechanisms in order to better understand the relationship between ICIs and the development of CD and CD-like alterations, including the histological diagnostic approach.

## 3. Materials and Methods

A systematic review was conducted following the preferred reporting items for systematic reviews and meta-analyses (PRISMA) guidelines. A search of PubMed and Web of Sciences (WoS) databases was performed up to 30 September 2021 using the terms: immune checkpoint, immune checkpoint inhibitor, imatinib, pembrolizumab, nivolumab, atezolizumab, avelumab, and durvalumab in combination with each of the following: celiac, coeliac, celiac-like, sprue-like, sprue like, duodenitis, enteropathy. Only articles in English were selected.

All original reports in which the development of CD or CD-like conditions were documented histologically in patients of any age being treated with ICIs were considered for inclusion. Only articles in English were selected. Case reports, letters to the editor, and original articles were also included.

## 4. Results

Based on our predefined inclusion criteria, the literature search identified ten papers in the period 2013–2021. While two of them were original papers, the remaining ones were either case reports or letters to the editor [33,50,51,52,53,54,55,56,57,58]. Overall, twenty-five patients were described in these publications with eleven of them receiving a diagnosis of CD confirmed by serological and histological assessment. These data are summarized in Table 1. Regarding CD patients, a genetic evaluation for DQ2 and DQ8 was reported only in one case [50], EMA was tested in two cases [50,57], TTGA IgA class in ten cases [33,50,53,55,56], and TTGA of IgG class in one case [57] (Table 1).

**Figure 1:**
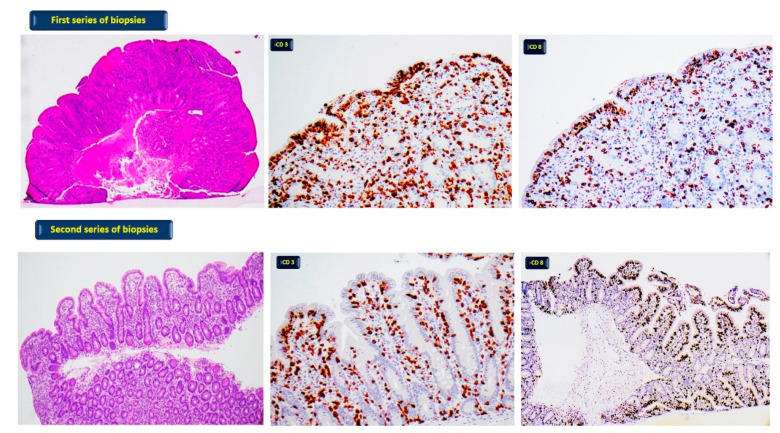
First series of biopsies: patient without GFD; diffuse moderate-severe atrophy of villi (haematoxylin-eosin 40×) with pathological increase of T lymphocytes CD3 (400×) and CD8 positive (400×). Second series of biopsies: patient on GFD; normal villi with focal low atrophy (haematoxylin- eosin 100×) and pathological increase of T lymphocytes CD3 (400×) and CD8 positive (100×).

Among the reported treatments, pembrolizumab appears to be the most frequent one associated with CD, even after months of treatment initiation. All ICIs-CD cases reported so far have shown typical CD histological findings with variable villous atrophy and intraepithelial lymphocytosis. The clinical presentation of affected patients was mostly characterized by diarrhoea, frequently associated with weight loss. Interestingly, patients diagnosed with ICIs-CD benefited from a gluten-free diet (GFD). In two cases, steroid treatment was also implemented. In addition, four case reports of ICIs-related CD-like conditions have been described, but CD serologic testing was negative and, of note, only one of them showed clinical benefits from the introduction of a GFD. We recently observed the case of a 45-year-old man treated with ipilimumab for follicular lymphoma. Four weeks after initiating the therapy, the patient developed non bloody diarrhoea and weight loss. The duodenal biopsies showed a picture of an ICIs-related CD-like condition characterized by a moderate-severe atrophy of villi and intraepithelial lymphocytosis. After a GFD, the duodenal biopsies were performed again, and we observed improvement in villous architecture but persisting intraepithelial lymphocytosis (Figure 1).

## 5. Discussion

In recent years, the continuously wider implementation of ICIs in oncology has increased the occurrence of IRAEs. Regarding events arising in the gastrointestinal tract, CD and CD-like conditions have rarely been described and their real incidence among ICIs-related IRAEs is unknown, with only a very small number of cases reported to date. On the other hand, ICIs-related duodenitis and enteritis appear more frequent. The involvement of the upper gastrointestinal tract is more common among patients treated with anti-PD1/PDL-1 agents compared to patients in treatment with anti-CTLA4 agents, in which the endoscopic and histological injuries are detected more often in the stomach than in the duodenum [59]. The most common endoscopic abnormalities reported in the duodenum are erythema, erosions, substenosis, ulcers, mucosal flattening [32]. There is no correlation between the endoscopic abnormalities and histological lesions; in fact, histological lesions are found in endoscopically normal mucosa and biopsies are indispensable for the diagnosis of ICIs injuries [24,32,59]. The spectrum of histological lesions that can be observed in the duodenal and intestinal mucosa during ICIs therapy is broad [32,60,61,62,63,64], including expansion of the lamina propria by lymphoplasmacytic infiltrate accompanied by neutrophils and eosinophils, a variable degree of villous atrophy (subtotal or total) with patchy intraepithelial lymphocytosis, occasional increases in epithelial cryptal apoptosis, and non-necrotizing granuloma unassociated with crypt rupture [23,24,32]. The type of therapeutic agent seems to influence the histologic appearance. The intraepithelial lymphocytosis was more frequently observed in patients in anti-PD1 therapy [32] and this feature appears to be a key component in pathogenesis of PD1 IRAEs [63]. On the contrary, Gonzalez et al. [64] found in their PD-1 inhibitor cohort neutrophilic villitis in absence of prominent intraepithelial lymphocytosis and apoptosis. The histologic pattern largely reported is duodenitis with irregular villous pseudo-atrophy. Irshaid et al. recently described in detail the histological and immunophenotypic features of ICIs-related gastritis and duodenitis [30]. While histology of ICIs-related gastritis overlapped with that related to *Helicobacter pylori* infection, the histological presentation of duodenitis was similar to the classical CD but, in our opinion, duodenitis is a misnomer and the ICIs-related injury of the duodenal mucosa should more correctly be defined as CD-like enteropathy. In detail, the histological clue of ICIs-related duodenal injury is represented by a marked villous atrophy (from moderate to severe) with a mechanism similar to olmesartan, and increased intraepithelial lymphocytic infiltration; when compared to classical CD, ICIs-related CD-like conditions show an increased presence in the lamina propria of CD3+ and CD8+ T lymphocytes, with a reduced CD4:CD8 ratio [30].

Thus, ICIs can cause atrophic lesions of the intestinal mucosa with CD-like features that are difficult to differentiate from classical CD. Subtle histological change such as patchy intraepithelial lymphocytosis [32] or more prominent eosinophil and neutrophil infiltration in the lamina propria, and more erosions than in classical CD [30,42] can suggest a diagnosis of ICI-related CD-like conditions. For an accurate differential diagnosis between these two entities, serological data are necessary. Autoimmune enteropathy, Crohn’s disease, peptic duodenitis involving the duodenal bulb, nonsteroidal drugs abuse, olmesartan, infection (viral gastroenteritis) must be also considered in the differential diagnosis [24,30]. In particular, autoimmune enteropathy, characterized by a marked villous atrophy, intraepithelial lymphocytosis and apoptotic cryptal bodies must be distinguished from this CD-like condition [24,30].

About etiology in CD-like lesions, Freeman et al. [65] suggested that ICIs may trigger a type of enteritis similar to the olmesartan-associated sprue-like enteropathy (OAE), as well as other angiotensin II receptor antagonists [44]. ICIs-related enteropathy, such as OAE, mimics CD from a clinical and histological point of view but lacks the presence of serum TTGA. Moreover, ICIs-related enteropathy can cause simultaneous involvement of the lower and upper gastrointestinal tract and, similarly to OAE, appears to be a “multifaced” histological entity [66].

Concerning ICIs-CD, since 2013, only eleven cases have been reported with histological documentation in the literature [33,53,55,56,57]. The serological data reported are often incomplete. Of interest, while clinical and morphological features are similar between ICIs-CD and classical CD [33], the serological and immunophenotypic profiles appear to be rather different. In fact, ICIs-CD is characterized by the positivity for IgA TTGA without IgA EMA (these data are reported only in two cases), which are positive in the vast majority of classical CD patients [67]. In the study by Badran et al. [33], TTGA titres in ICIs-CD are reported to be very high with a range from 104 to >300 IU/mL, so this swept aside any suspicion of a false positive TTGA result which is confined to very low antibody titres [67]. It must be underlined that the diagnosis of ICIs-CD remains doubtful in the two patients showing a very low titre positivity for IgA TTGA and for IgG TTGA [53,57], due to the low specificity of these antibody patterns. Moreover, with regard to immunophenotypic profile, classical CD shows increased intraepithelial CD3+ and CD8+ T cells with a marked increase in intraepithelial γδ T cells compared to ICIs-CD [68]. Furthermore, ICIs-CD has an increased presence of CD68+ and PD-L1+ macrophages in the lamina propria when compared to classical CD. As for genetic profiles, only one of ICIs-CD cases was tested for HLA-DQ2 and -DQ8 and was found to be positive for the typical celiac pattern [50]. This is a limit of the previous studies on this topic, since the absence of these haplotypes excludes the diagnosis of CD. Interestingly enough, a slight prevalence of male gender characterizes ICIs-CD (M/F:1.2), whereas female gender is a lot more frequent in classical CD (F/M:3.0) [67].

These histological and immunological differences, along with clinical improvement after GFD and a reported increased serological value of autoantibodies in ICIs-CD, suggest that the onset of this entity may have a distinct immune mechanism which in turn is triggered by the implementation of the same drugs. Conversely, though ICIs-related CD-like enteropathy share the same histological and immunophenotypic characteristics with ICIs-CD, the lack of clinical improvement after GFD and the negativity for IgA TTGA suggest a different immune mechanism. Moreover, since ICIs-CD improved with GFD, similarly to the classic CD, it is likely that gluten becomes a trigger for these forms as well, upon treatment initiation [33]. Finally, steroid-based therapy was needed only in ICIs-CD cases associated with gastritis and colitis [50,53].

While it is well known that ICIs may trigger gastrointestinal inflammatory responses, the underlying mechanisms are poorly understood. The pathogenesis appears to be multifactorial and may involve the activation of effector T cells, increasing memory T cells, and lymphocytes in the intestinal mucosa, which may interact with the resident microbiota, infectious triggers, and drugs [65]. In addition, it remains to be established whether the ICIs-CD arises on latent pre-existing CD exposed by using these compounds or, instead, whether it represents a CD arising de novo [33].

Finally, ICIs can induce intraepithelial lymphocytosis with villous atrophy in the intestinal mucosa. These lesions can be the expression of a drug-induced damage or of a latent CD. In fact, ICIs do not cause CD but promote the appearance of a latent CD through the loss of immunological tolerance [33].

The limitations of our conclusions are due to the few papers, case reports, and case series that are reported in the literature and an absence of controlled studies. Furthermore, the papers published are lacking relevant data (serology and genetics) useful for validating a correct diagnosis of CD.

## 6. Conclusions

ICIs can induce enteropathy and mucosal sampling for histological evaluation is mandatory. In the case of morphological lesions resembling CD, serological and genetic testing should be performed for differentiating true CD from drug-induced villous atrophy. This diagnostic path must be as more accurate as possible, including both IgA TTGA and EMA as well as HLA-Q2 and DQ8 typing. Moreover, in this setting, the diagnosis of CD should be made with caution and after a thorough evaluation because the therapeutic approach to ICIs-CD (GFD) differs from ICIs-related CD-like conditions (corticosteroids).

## Figures and Tables

**Table 1:** Studies reporting cases of celiac disease and celiac like conditions related to ICIs therapy.

Author, Year	No. Cases Histological Documented	Sex Age	Drugs	Duration Exposure ICIs	HLA DQ2 or DQ8	TTGA U/mL IgA	EMA	Villous Atrophy	IELs	Diagnosis	Others GI Findings	Therapy
Gentile et al. [50], 2013	1	M 62	Ipilimumab	6 weeks	Pos	79.1	Neg	Partial/Total	60/100	ICIs-CD	Colon apoptosis	GFD Budesonide
Facchinetti et al. [51], 2018	1	F 42	Nivolumab	Several months	Neg	Neg	Neg	Total	˃25/100	CD-like	Collagenous colitis	Budesonide
Duval et al. [52], 2019	1	M 58	Nivolumab	1 month	NR	Neg	NR	Subtotal	High number	CD-like	Absent	Methylprednisolone
Alsaadi et al. [53], 2019	1	F 74	Ipilimumab+Nivolumab	1 week	NR	12	NR	Total	20–30/100	ICIs-CD	Active chronic gastritis	GFD Budesonide
Kokorian et al. [54], 2019	1	M 65	Nivolumab	13 months	NR	Neg	Neg	Subtotal	40/100	CD-like	Absent	Prednisolone
Arnouk et al. [55], 2019	1	M 79	Pembrolizumab	1 week	NR	59	NR	Total	˃25/100	ICIs-CD	Absent	GFD
Badran et al. [33], 2020	6	M 44-73	NR	82.5 days	NR	121.21 ± 80.29	NR	Moderate-severe	25(±11)/ 100	ICIs-CD	Absent	GFD
Schoenfeld et al. [56], 2020	1	F 72	Pembrolizumab	Five cycles	NR	37	NR	NR	Increased	ICIs-CD	Absent	GFD
Sethi et al. [57], 2021	1	F 63	Pembrolizumab	Few months	NR	5/IgG	Neg	Total	Increased	ICIs-CD	Absent	GFD
Theodoraki et al. [58], 2021	1	M 51	Pembrolizumab	6 months	NR	Neg	Neg	Present	Present	CD-like	Lymphocytic colitis	GFD

ICIs-CD: celiac disease arising during therapy with immune checkpoint inhibitors; NR: not reported; TTGA: tansglutaminase antibodies; EMA: antiendomysial antibodies; IELs: intraepithelial lymphocytes/100 enterocytes; CD: celiac disease; GI: gastrointestinal; GFD: gluten-free diet. Badran et al. [33] and Schoenfel et al. [56] reported 8 and 9 cases but only 6 and 1 cases, respectively, had histological diagnostic confirmation.
